# A quality-by-design eco-friendly UV-HPLC method for the determination of four drugs used to treat symptoms of common cold and COVID-19

**DOI:** 10.1038/s41598-023-28737-3

**Published:** 2023-01-28

**Authors:** Nora A. Abdallah, Mona E. Fathy, Manar M. Tolba, Amina M. El-Brashy, Fawzia A. Ibrahim

**Affiliations:** Department of Pharmaceutical Analytical Chemistry, Faculty of Pharmacy, Mansoura University, Mansoura, 35516 Egypt

**Keywords:** Analytical chemistry, Environmental chemistry, Green chemistry

## Abstract

An optimization approach based on full factorial design was employed for developing an HPLC–UV method for simultaneous determination of a quaternary mixture used for the treatment of symptoms related to common cold and COVID-19. The quaternary mixture is composed of paracetamol, levocetirizine dihydrochloride, phenylephrine hydrochloride and ambroxol hydrochloride. The developed technique is a green, fast and simple method that uses isocratic elution of mobile phase consisting of 20:5:75 (v/v) of ethanol: acetonitrile: 2.5 mM heptane-1-sulphonic acid sodium salt at pH 6.5 $\pm$ 0.02. The chromatographic separation was carried out using Hypersil BDS Cyano LC Column (250 × 4.6 mm, 5 μm) with 230 nm UV detection and 1.0 mL/min. flow rate. Avoiding the routine methodology and resorting to the modern technology—represented in the usage of experimental design—allows rapid determination of the four drugs using the optimum quantity of chemicals to avoid any waste of resources. The quaternary mixture was eluted in less than 9 min., where retention times of paracetamol, levocetirizine dihydrochloride, phenylephrine hydrochloride and ambroxol hydrochloride were found to be 2.2, 3.8, 6.6 and 8.8 min., respectively. The calibration graphs of the four drugs were linear over concentration ranges of 50.0–500.0, 0.5–20.0, 0.5–20.0 and 0.5–100.0 µg/mL for paracetamol, levocetirizine dihydrochloride, phenylephrine hydrochloride and ambroxol hydrochloride, respectively with correlation coefficients higher than 0.999. The method is accurate with mean recoveries between 99.87 and 100.04%, precise, as %RSD for the intraday and interday precision were between 0.61 and 1.64% and very sensitive with limit of detections (LOD)’s between 29 and 147 ng/mL and limit of quantification (LOQ)’s between 95 and 485 ng/mL. The proposed method was successfully applied for the analysis of the four drugs either in raw materials or in prepared tablet with the least amount of chemicals within short time. It is also validated following International Conference on Harmonization Guidelines. The proposed method was found to be green according to the most common greenness assessment tools; NEMI, GAPI, Analytical Eco-Scale and AGREE methods. The advantages of the proposed method qualify it for routine analysis of the studied drugs either in single or co-formulated dosage form in quality control labs.

## Introduction

The common cold has attracted the public and physicians for centuries. It has been defined as an acute epidemic respiratory disease characterized by mild coryzal symptoms of rhinorrhea, nasal obstruction, and sneezing. The disease is self-cured, but the symptoms may persist for 2 days to more than 14 days. The symptoms represented in fever, cough, sore throat, or lacrimation^1^. The common cold is the most frequent acute illness, as about half the population gets at least one cold every year^2^. COVID-19 induces shortness of breath, cough, fever, nasal congestion and general malaise^3^. To date, there are no antiviral medications that can fight the viruses that cause the common cold. But that does not necessarily mean to suffer with each and every symptom until the body fights off the virus, as it can be treated symptomatically using over the counter analgesics, antihistamines, cough suppressants and decongestants.

Paracetamol (PAR), levocetirizine dihydrochloride (LVC), phenylephrine hydrochloride (PHN) and ambroxol hydrochloride (AMB) are used as first line to relief the symptoms of the common cold or the COVID -19. The four drugs are combined in one tablet called Cheston Cold Total tablet which is widely used in India. It is mainly used for the prevention or controlling of common cold symptoms^4^. Taking unit dosage form (tablet) instead of four (2 tablets and 2 capsules) is more convenient for the patients and need less preparation steps providing economic and environmental benefits.

**Figure 1:**
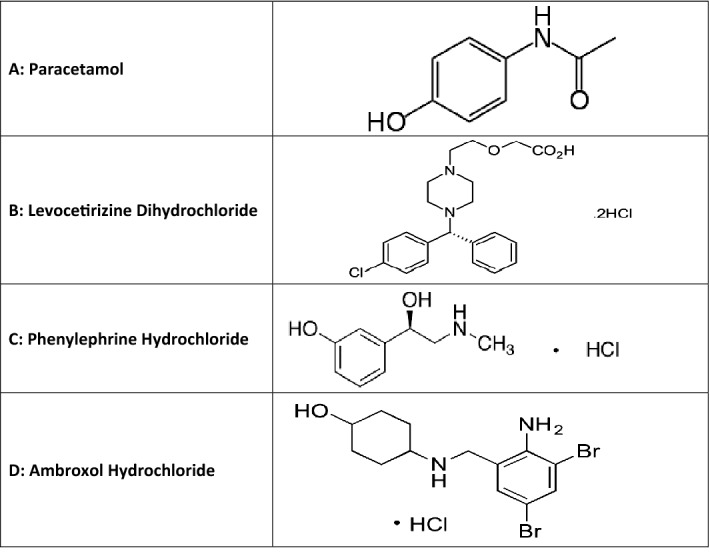
Chemical structure of (**A**) paracetamol, (**B**) levocetirizine dihydrochloride, (**C**) phenylephrine hydrochloride, (**D**) ambroxol hydrochloride.

Paracetamol (PAR); Fig. 1A, is a para-aminophenol derivative which has analgesic, antipyretic effect and weak anti-inflammatory activity. Paracetamol is administered for relieving mild to moderate pain and as antipyretic for short-term treatment. Usually, PAR is the chosen analgesic or antipyretic, mainly in old patients and patients who cannot take salicylates or other NSAIDs, such as children, patients who have asthma or previously diagnosed with peptic ulcer. It is official in United States Pharmacopeia (USP)^5^, British Pharmacopeia (BP)^6^ and Japanese pharmacopeia (JP)^7^ and it was assayed by RP HPLC in both USP and BP and via spectrophotometry in JP.

Levocetirizine dihydrochloride (LVC); Fig. 1B, is chemically designed as 2-(2-{4-[(R)-(4-Chlorophenyl)(phenyl)methyl]piperazin-1-yl)ethoxy)acetic acid ^8^. It is given orally to children for the symptomatic relief of allergic rhinitis and chronic urticaria^9^. It is usually combined with analgesics and decongestant for treatment of common cold symptoms. It is also a non-sedating antihistamine as it does not cross the brain barrier and therefore is unlikely to cause drowsiness^10^. It is official in USP^5^ and assayed by HPLC.

Phenylephrine hydrochloride (PHN); Fig. 1C, is chemically -[(1R)-1-Hydroxy-2-(methylamino)ethyl]phenol.HCl^11^. It is a sympathomimetic with mainly direct effects on adrenergic receptors. It has mainly alpha-adrenergic activity without significantly stimulating effects on the CNS at usual doses. It is most commonly used by mouth for the symptomatic relief of nasal congestion. It is often included in preparations intended for the relief of cough and cold symptoms^9^. It is official in both USP^5^ as well as BP^6^ and assayed in them using voltammetry and potentiometric titration, respectively.

Ambroxol hydrochloride (AMB); Fig. 1D, is chemically designed as trans-4-(2-Amino-3,5-dibrombenzylamino)-cyclohexanol ^12^. It breaks up the mucous produced by the respiratory system allowing patients to breathe freely and deeply. It is often administered as an active ingredient in cough syrup^13^. It is also used to relief pain in acute sore throat related to pharyngitis caused by viral infection^14^. It is official in European Pharmacopeia (EP) and assayed in it through potentiometric titration^15^.

Various analytical methods using HPLC were reported for determination of some drugs that are used for treatment of one or more cold symptoms. Two anti-histaminic agents, diethylcarbamazine and levocetirizine, were assayed in its tablet formulation by reverse phase high performance liquid chromatography^16^. A combination of Salbutamol sulfate, Guaifenesin, and Ambroxol hydrochloride that are used for suppression of cough was assayed in combined tablet dosage form using HPLC^17^. HPLC–DAD method was reported for the analysis of a complex mixture consisting of phenylephrine hydrochloride, paracetamol, caffeine and levocetirizine^18^. Gradient RP-HPLC method was developed for the simultaneous estimation of ascorbic acid, phenylephrine HCl, paracetamol, levocetirizine HCl in tablet dosage form^19^. Three decongestant drugs, chlorpheniramine maleate, paracetamol and phenylephrine hydrochloride were quantitatively determined in pharmaceutical preparations using HPLC^20^. Only three analytical procedures were reported for the determination of the studied quaternary mixture in the pharmaceutical preparation. These are: First-order derivative spectrophotometry^21^, gradient HPLC–DAD method^22^ and isocratic HPLC-UV^23^.

Most of the optimized separation methods cited in the literature for the analysis of common cold drugs by RP-HPLC involve studying of a large number of variables in the separation process. In addition, those methods use large quantities of organic solvent in the mobile phase which produce negative effect on the environment. For this reason, it is needed to design a more effective, green and time-saving method using the experimental design procedure.

To the best of our knowledge, no research involving full factorial design experiment, for the separation of those drugs, was reported. Experimental design (DOE) has a lot of advantages in optimizing and development of methods based on statistical analysis. A full factorial design (FFD) is a type of DOE ‘multivariate optimization’ which allows investigating the effect of all the factors simultaneously based on the responses of the dependent factors and the interactions between the independent factors^24^.

The concept of green analytical chemistry has a great scientific interest in the last few years. Green analytical chemistry aims at lessening or removing harmful chemicals used in analytical techniques, reduction in energy consumption^25^ and minimization of waste production^26^, without affecting the analytical performance of the method^27^. Liquid chromatography is one of the most harmful techniques, as it uses high amounts of harmful organic solvents^28^. So, one of the main targets of greening chromatography is a solvent-reducing approach, where the most common mobile phases containing methanol are replaced by less harmful and more ecofriendly alternatives, such as water, ethanol and isopropanol^29^.

The aim of this newly developed method is to develop a full factorial designed, greener, more sensitive and faster method for estimation of the quaternary mixture either in raw material or dosage form with no interferences of the dosage form additives. The developed method was designed using FFD to ensure using the optimum amount of chemicals and obtaining optimal performance and reliability of the used parameters and the results of the proposed method^30^. The developed method employs a Cyano column and an UV detector which is characterized by its signal stability and availability in most labs. The elution was isocratic using a mobile phase consisting of small percentage of organic solvents, 20:5:75 (v/v) of ethanol: acetonitrile: 2.5 mM heptane-1-sulphonic acid sodium salt (HSA) at pH 6.5 $\pm$ 0.02. The designed method was successfully applied in the analysis of the four drugs either in laboratory prepared synthetic mixtures, the laboratory prepared tablets or dosage forms. This method was fully validated according to the International Conference on Harmonization (ICH) validations guidelines^31^. The greenness profile of the proposed method was performed adopting four assessment tools. The analytical performance, results and the greenness profile of the proposed method were compared with those of the reported methods. The analytical performance and the greenness profile of the proposed method was found to be greener, more sensitive and economical than the reported HPLC methods^22,23^.

## Experimental design; materials and method

This section contains the information about all the used equipment, software, chemicals and reagents. It also describes the steps for preparing the samples to be analyzed by the proposed method.

### Equipment

- Knauer Chromatograph equipped with a Knauer, D-14163 injector valve with a 20 µL loop (Berlin, Germany).
- Eluent was filtered using 0.45 µm membrane filters (Millipore, Cork, Ireland).
- Consort NV P-901 calibrated pH–Meter (Belgium) was used for pH measurements.
- Dissolution was done by Digital Ultrasonic Cleaner, Model: Soner 206 H, MTI Corporation (USA).

### Software

Factorial design statistical analysis was performed using Minitab 16.2.0, USA.

### Chemicals and reagents

- Raw forms of the PAR and AMB were obtained from Glaxosmithkline in Egypt, LVC was obtained from Marcyrl Pharmaceutical Industries in Egypt, and PHN was from October pharma in Egypt.
- Prepared tablets contain 325 mg PAR, 5 mg LVC, 5 mg PHN and 30 mg AMB, prepared at Pharmaceutical Analytical Chemistry lab, Faculty of Pharmacy, Mansoura University.
- Carlo Erba Ethanol absolute anhydrous ≥ 99.9%, Emmendingen, Germany.
- Heptane-1-sulphonic acid sodium salt 99% (HSA), United Kingdom.
- Acetonitrile HPLC grade, Fisher Scientific Co., USA.
- Panadol Advance, 500 mg PAR/tablet (Batch no. 1140103), Levcet, 5 mg LVC/ tablet (, Batch no. 2132195), Ambroxol, 30 mg AMB/tablet (Batch no. 242925 A) were purchased from a local Egyptian pharmacy.

### Standard solutions preparation

1.0 mg/mL of PAR and 200 μg/mL of the rest of the drugs were prepared separately in 50 mL volumetric flasks by dissolving 50 mg of PAR and 10 mg of the raw materials of the rest of the drugs in ethanol. Working standard solutions were prepared on demand by further dilution of different volumes of the stocks with mobile phase. All solutions were found to be stable upon storage in the refrigerator for about three weeks.

### Procedures

The steps for preparing the raw material samples for analysis to obtain the regression equation is described below. The procedures for preparing synthetic mixtures, the prepared tablet or extracting the studied drugs from their dosage forms are also explained in detail.

#### Calibration graphs construction

Different volumes of each drug standard solution were transferred to four sets of 10 mL volumetric flasks. Mobile phase was used to complete the flasks to their marks. The produced concentrations covered the concentration ranges of 50.0–500.0, 0.5–20.0, 0.5–20.0 and 0.5–100.0 μg/mL for PAR, LVC, PHN and AMB, respectively. 20 μL of each concentration was injected three times and eluted using the optimum chromatographic conditions. Average peak areas for each drug was recorded and plotted against its corresponding concentrations to create the calibration graph and regression equation for each of the four drugs.

#### Analysis of the studied drugs in synthetic mixture

Different aliquots of the stock solutions were moved to a set of 10 mL flasks achieving the ratio of Cheston cold tablets^32^ as 65:1:1:6 of PAR: LVC: PHN: AMB. The flasks were completed with the mobile phase, then the previously mentioned procedure under Section “Calibration graphs construction” was followed. The percent found was calculated from the corresponding regression equation.

#### Analysis of the four drugs in their co-formulated prepared tablet

650 mg, 10 mg, 10 mg and 60 mg of PAR, LVC, PHN and AMB, respectively raw powders were mixed with 20 mg of talc powder and 15 mg of maize starch, lactose, glucose and fructose to prepare two tablets. An accurately weighted amount of the prepared tablets equivalent to 325 mg of PAR, 5 mg of both LVC and PHN in addition to 30 mg of AMB was dissolved in 60 mL of mobile phase in 100 mL volumetric flask. The volumetric flask with its contents was subjected to sonication for 30 min. followed by addition of the mobile phase till complete volume. After that, filtration was performed. Different concentrations inside the working range of each drug were prepared and the previously explained method in “Calibration graphs construction” section was followed. The content of each drug in the prepared dosage form was calculated using the corresponding regression equation.

#### Analysis of the PAR, LVC and AMB drugs in their single dosage forms

Ten Panadol advance (500 mg) or Levcet (5 mg) or Ambroxol (30 mg) tablets were finely ground individually. Accurately measured amount of each powder was transferred into 100 mL measuring flask. 60 mL ethanol was added to each flask followed by sonication for 30 min., then ethanol was added to reach the flask mark. After filtration was carried out to remove insoluble excipients, sequential concentrations were prepared as required to achieve the working ranges of the studied drugs. Finally, the previously explained method in “Calibration graphs construction” was followed. The content of each drug in the prepared dosage form was calculated using the corresponding regression equation.

#### Experimental design

**Figure 2:**
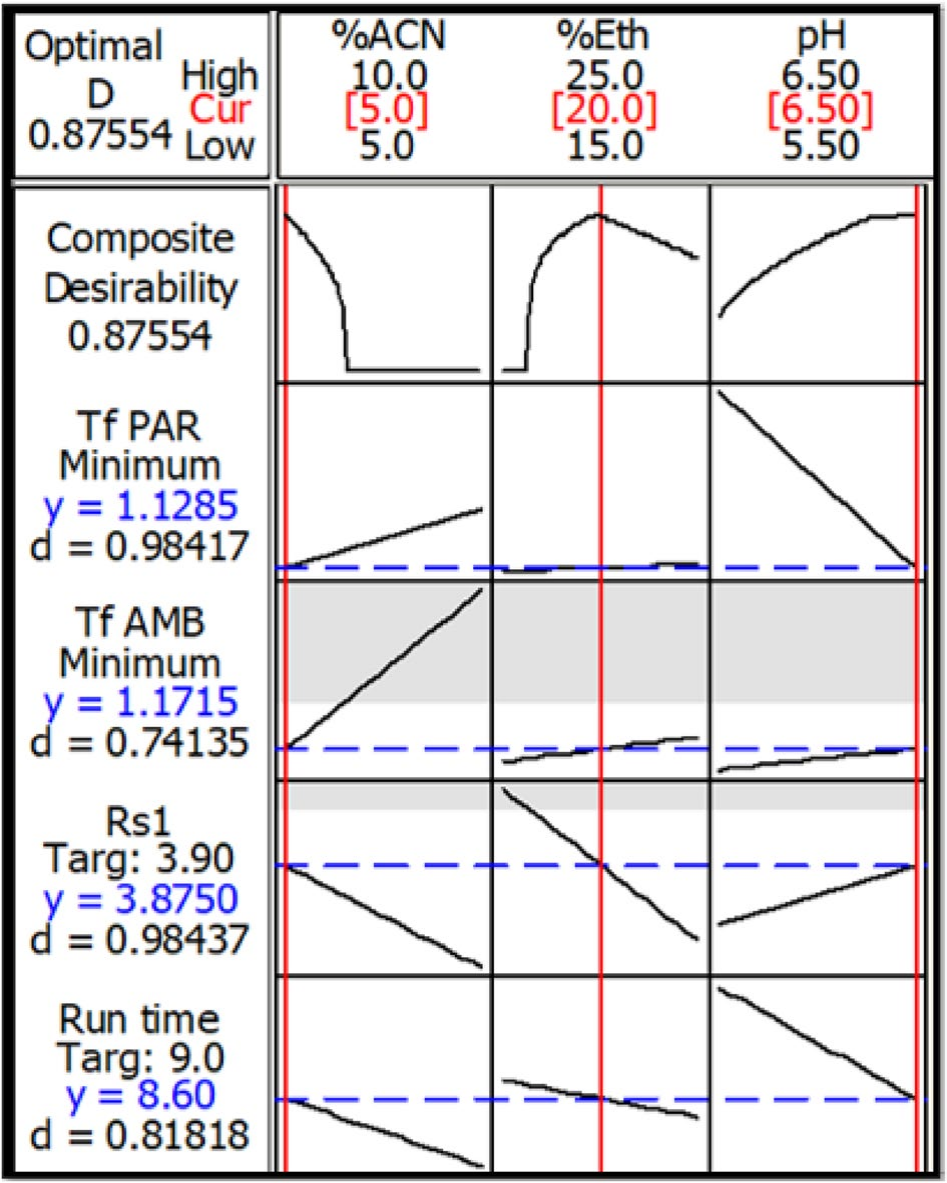
2^3^ full factorial design (FFD) optimization plot.

Experimental design is a process that depends mainly on making systematic plans that make full use of minimum experimentation to obtain maximum information then employing it using statistical models to make significant conclusions from the obtained results^33^. Multilevel factorial design, 2^3^ FFD was applied in this study for the determination of optimal conditions that produced the ideal response values. Minitab optimizer is provided with upper, target, and lower values for each response, then calculates the optimum conditions and use them to draw a plot, as shown in Fig. 2. The optimization plot shows the effect of each parameter on the responses and chooses the optimum of each factor for best responses. All details of how to carry out DOE process and how it calculated the optimum conditions are explained in detail in EL-Shorbagy et al.^34^.

## Results and discussion

**Figure 3:**
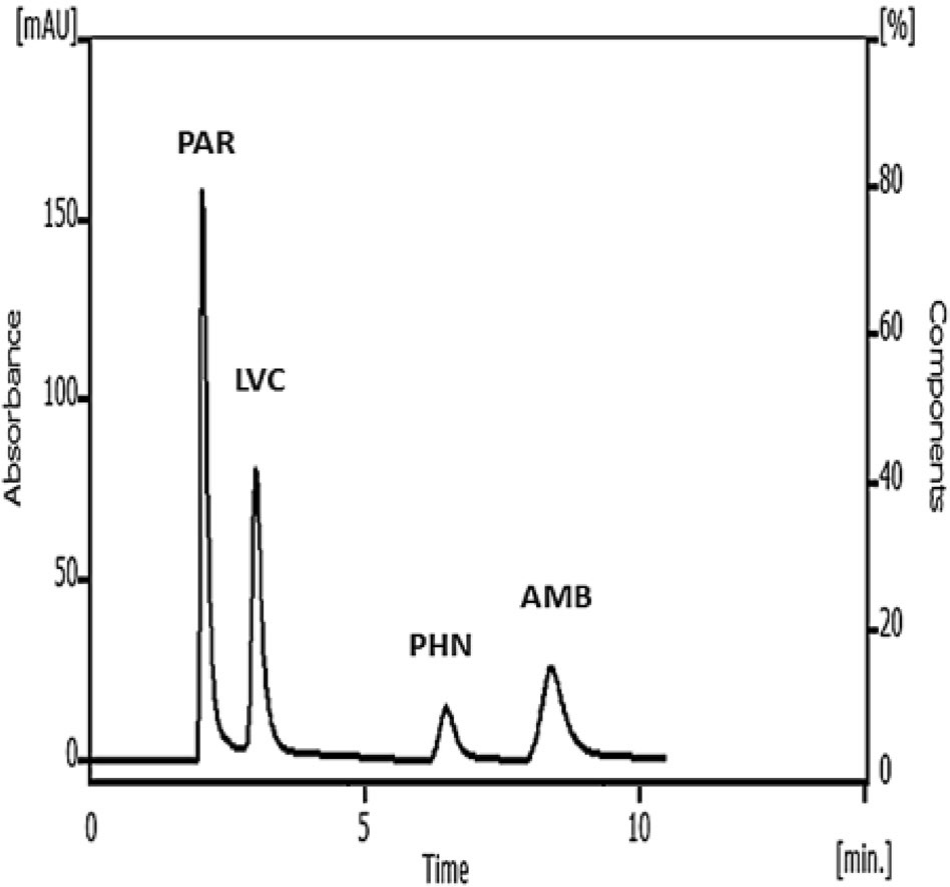
Typical chromatogram of PAR, LVC, PHN and AMB mixture (50.0 µg/mL each drug) under the described chromatographic conditions.

The proposed method presents a green, fast, sensitive and economic RP-HPLC technique for resolving a quaternary mixture used to relief symptoms associated with common cold or COVID-19. The proposed technique employs factorial design to optimize and maintain the optimum parameters used for separation; hence it saves time and resources. The four drugs were well resolved and separated using isocratic elution of an aqueous mobile phase consisting of small volume of ethanol, very small amount of acetonitrile and HSA as an ion pairing reagent in less than 9 min. Several parameters were investigated for the sake of obtaining the optimized ratios of the mobile phase and suitable column to produce good separation. Utilizing of 2^3^ FFD ensured the reliability and optimal performance of the method without wasting any extra solvents or chemicals. Good optimization led to decreasing the environmental hazards through the usage of eco-friendly and relatively safe solvent, such as ethanol. The optimization also resulted in shrinking the required time for chromatographic analysis and consequently reducing waste production, while maintaining the best resolution and sensitivity. Typical chromatogram of symmetrical peaks of synthetic mixture of PAR, LVC, PHN and AMB is shown in Fig. 3. The response optimization of 2^3^ full factorial design for the separation of the studied drugs is shown in Table 1.

**Table 1:** Response optimization of 2^3^ full factorial design for HPLC–UV separation of quaternary mixture.

Factor	Goal	Lower	Target	Upper	Weight	Import	Predicted responses	Desirability
T_f_ PAR	Minimize	1.1	1.1	2.9	1	1	1.1285	0.98417
T_f_ AMB	Minimize	1.0	1.02	1.7	1	1	1.1715	0.74135
Rs _1_	Target	2.3	3.9	4.6	1	1	3.8750	0.98437
Run Time	Target	6.8	9	11.1	1	1	8.60	0.81818
Optimum conditions: 20% ethanol, 5% Acetonitrile and pH 6.5							Composite desirability (D) = 0.87554	

### Method optimization

All the parameters affecting the proposed method were investigated to know the most suitable parameters. The most suitable parameters were employed to analysis the studied drugs.

#### Optimization of the chromatographic conditions (screening experiment)

Well symmetrical peaks with good resolution were obtained after several trials and summarized under the following subheadings.

##### UV wavelength selection

**Figure 4:**
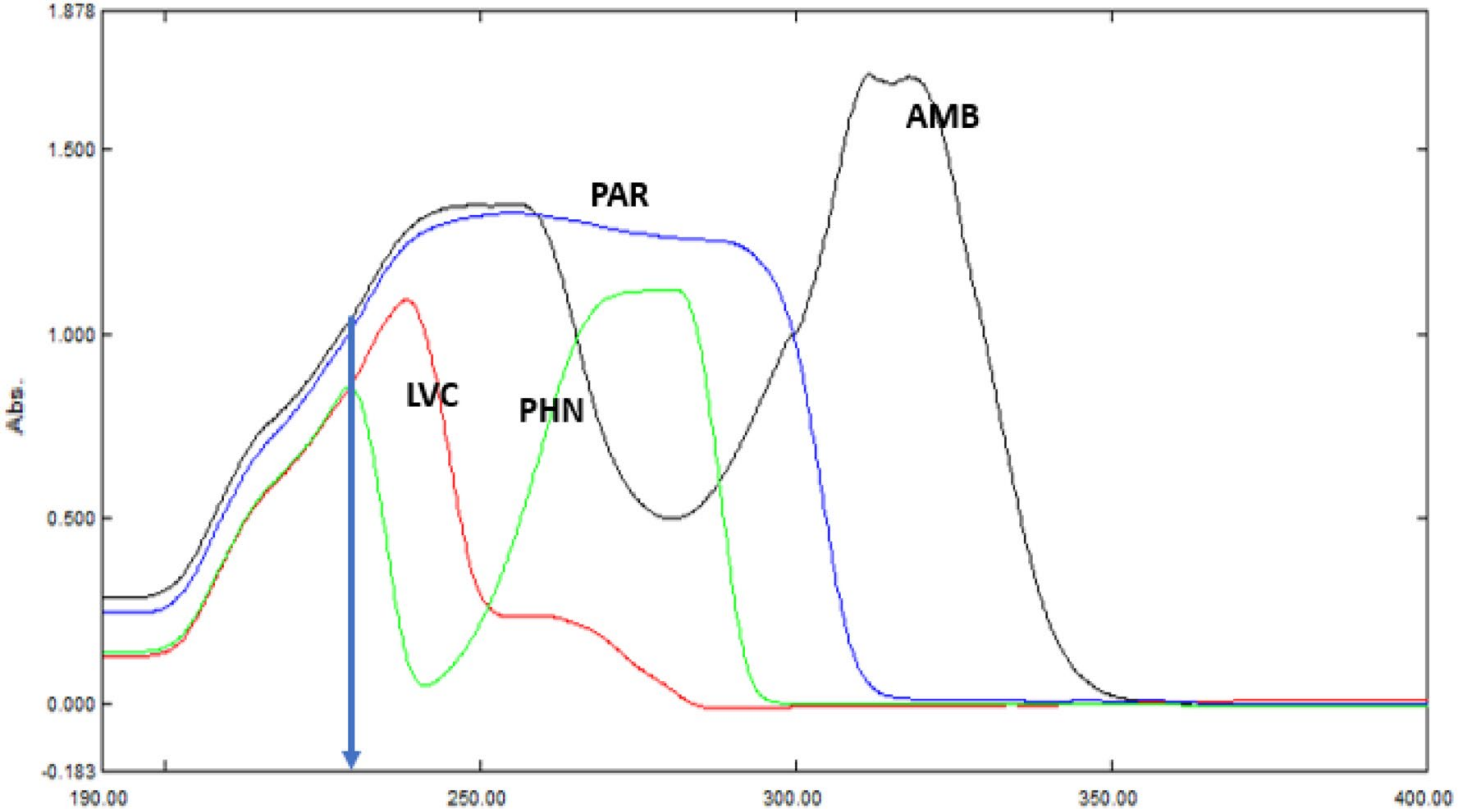
Absorption spectra of PAR, LVC, PHN and AMB.

To choose the most suitable wavelength, the four drugs dissolved in ethanol were scanned separately by spectrophotometer. Figure 4 shows that 230 nm is the most suitable wavelength attaining high sensitivity of the four drugs. It also did not conflict with the fact that the analysis wavelength needs to be 20 nm above the used mobile phase UV cutoff, 210 nm for ethanol and 190 nm for acetonitrile^35^, to decrease the background noises during baseline stabilization stage^34^.

##### Column

Three columns were tried to choose the one that achieves the best resolution of the four drugs using small amount of the organic solvents. These columns included Hypersil BDS Cyano LC column (250 × 4.6 mm, 5 μm), Thermo scientific Hypersil C8 column (150 mm × 4.6 mm i.d., 5-µm) and Thermo scientific Hypersil C18 column (150 mm × 4.6 mm i.d., 5-µm). The experimental trials showed that the last two columns failed to separate the quaternary mixture with acceptable resolution, while the first one succeeded to separate them in symmetrical, well resolved and defined peaks. The Cyano column was considered an ideal choice for separating PAR, LVC and PHN as they are highly polar analytes and have very close Log P. Log P was 0.46 for PAR ^36^, 0.87 for LVC ^37^ and − 0.40 for PHN^38^). It also can tolerate lower concentrations of organic modifier without any shift of retention^34^.

##### Mobile phase

This method was directed mostly to avoid the usage of hazardous solvents by utilizing ethanol as main organic solvent for RP-HPLC. Ethanol is believed to be a safe and less hazardous eluent, as it is distinguished by its high viscosity, low vapor pressure and consequently a less evaporation and inhalation potential, thus reducing the necessity of thorough waste cleaning. All these advantages give superiority to ethanol for usage in mobile phase^39^. PAR, LVC and PHN have very close log P which made them very hard to resolve with ethanol only, so a small amount of acetonitrile was added to decrease the mobile phase polarity^40^. Also, to help in separation of such overlapped peaks, HSA was added as an ion pairing reagent. Different concentrations (1.0–5.0 mM) of HSA were tried, only concentrations of 1.0–2.5 mM were effective in separating the overlapped peaks. The mobile phase pH was found to greatly affect the retention time (t_R_) of the studied drugs due to modulation of their ionization degree. Different pH values (3.0–7.0) were also tested to study their effect on the resolution of peaks. Separation only occurred when pH was from 5.0 to 6.5, as lower pH caused overlapping of LVC and PHN peaks. Flow rate from 0.8 to 1.2 mL/min. were tested to choose the best flow rate. Three independent factors were studied using experimental design to choose the most optimum conditions for resolving such a complicated mixture. These factors include pH values from 5.5 to 6.5 as well as different ratios of ethanol from 15 to 25% and acetonitrile from 5.0 to 10% v/v.

#### Experimental design

The main goal of experimental designs was to reach the optimum conditions with the minimum number of trials needed while examining the maximum number of factors. Some initial chromatographic experiments were required before performing an experimental design to determine the chromatographic factors which have significant effects on the chromatographic responses (screening experiment). In this experiment, three factors were found to affect the chromatographic performance including: % of organic modifier (ethanol and acetonitrile) and pH. These factors were mainly affecting the total run time, resolution between PAR and LVC and tailing factor of PAR and AMB, as shown in Table 1. From the previous experiments, 2^3^ full factorial design was applied for the current study optimization using two level combinations and three independent factors (pH and % of ethanol and acetonitrile). FFDs are the form of factorial designs in which all influencing independent factors (k) with (m) level combinations are investigated. The number of experimental runs needed for a FFD depends on the number of independent factors (k) to be studied. As a general rule, the design requires a total of m^k^ experiments^41^ to be performed. From the screening step, it was found that the optimum input ranges in the 2^3^ FFD design is as follows: organic modifier in the range of 15–20% for ethanol and 5–15% for acetonitrile, a pH in the range of 5.5–6.5. These critical factors were inserted into Minitab software to find their optimum conditions. The design suggested a set of 8 experiments are needed to represent interactions of the mentioned factors and their effects on selected chromatographic responses (run time, Rs between PAR and LVC and T_f_ of PAR and AMB). For the choice of the most critical factors influencing the method, a synthetic mixture containing 50 µg/mL of each drug was prepared. The suggested eight runs were carried out, then the obtained chromatograms were interpreted and the results were inserted into Minitab software to determine the dependent factors. Finally, response optimizer compromise between different responses then the optimum setting of the input variables and desirability values were concluded. According to the response optimizer and optimization plot (Fig. 2), it was found that the optimal chromatographic conditions were 20% v/v for ethanol, 5%v/v acetonitrile with a pH of 6.5. Pareto charts in Fig. S1 showed that the % ACN had the main effect on all dependent factors except for the T_f_ PAR. On the other hand, the pH was found to have the highest effect on T_f_ PAR. Figures S2 and S3 illustrate the interaction plots and the main effect plots of the three independent factors on the four dependent ones.

Finally, the suggested mobile phase composed of 20:5:75 (v/v/v) of ethanol, acetonitrile and 2.5 mM heptane-1-sulphonic acid sodium salt at pH 6.5 $\pm$ 0.02 using 1.0 mL/min. as a flow rate and UV detection at 230 nm. The suggested mobile phase was successful in resolving the four drugs with acceptable sensitivity.

## Suggested technique validity

To perform an appropriate evaluation, the proposed method was assessed for various validation parameters including, linearity, range, limit of quantitation (LOQ), limit of detection (LOD), precision, accuracy, and selectivity according to ICH Q_2_R_1_ recommendations^31^.

### Linearity, limit of quantitation (LOQ) and limit of detection (LOD)

The linearity of the developed method was investigated by treating the stock solutions of the studied analytes as outlined under “Calibration graphs construction” heading. The average of three replicate determinations was used for each result. Under the optimum chromatographic conditions, the linear ranges for quantification of PAR, LVC, PHN and AMB were studied. The ranges and the good linearity results were presented in Table 2. Statistical analysis of the produced data^42^ showed values of the coefficients of determination with small intercepts which proves good linearity of the calibration curves. Linear regression equations of PAR, LVC, PHN and AMB are as follow:

$${\text{AUP }} = \, - {14}.{633 } + { 31}.{4}0{\text{5 C}} \,\,\left( {{\text{r }} = \, 0.{9997}} \right)\,\, {\text{for PAR}}$$

$${\text{AUP }} = { 4}.{719 } + { 25}.{\text{567 C}}\,\, \left( {{\text{r }} = \, 0.{9999}} \right) \,\,{\text{for LVC}}$$

$${\text{AUP }} = { 2}.0{77 } + { 7}.{\text{484 C}}\,\, \left( {{\text{r }} = \, 0.{9999}} \right)\,\, {\text{for PHN}}$$

$${\text{AUP }} = { 2}.{37}0 \, + { 17}.{\text{331 C}}\,\, \left( {{\text{r }} = \, 0.{9999}} \right) \,\,{\text{for AMB}}$$

where AUP is the area under the peak, C is the concentration in µg/mL and r is the correlation coefficient.

**Table 2:** Analytical parameters and assay results for determination of the investigated drugs in pure form by the suggested and comparison method.

Drug	Linear range (μg/mL)	Slope	Intercept	r	% Found ± S.D	LOD	LOQ	Comparison method^23^	*t* and *F* tests (Tabulated values) at *P* = 0.05
PAR	50.0–500.0	31.405	− 14.633	0.9997	99.87 ± 1.23	0.029	0.095	100.22 ± 1.87	0.35 (2.30) 2.31 (5.14)
LVC	0.5–20.0	25.567	4.719	0.9999	99.65 ± 0.73	0.065	0.213	100.19 ± 1.49	0.77 (2.36) 4.28 (5.79)
PHN	0.5–20.0	7.484	2.077	0.9999	99.89 ± 1.27	0.147	0.485	100.11 ± 0.97	0.26 (2.36) 1.73 (19.29)
AMB	0.5–100.0	17.331	2.370	0.9999	100.04 ± 1.17	0.144	0.476	99.71 ± 1.93	0.35 (2.26) 2.69 (4.74)

The limits of detection and quantitation were estimated practically according to ICH Q2(R1) recommendations. A signal-to-noise ratio of 3:1 or 2:1 is considered acceptable as a general case for estimating LOD and a signal-to-noise ratio of 10:1 is considered acceptable for estimating the LOQ. The obtained results are summarized in Table 2. The obtained low LOD and LOQ indicate good sensitivity of the method.

### Accuracy

Statistical analysis was applied for comparison of the obtained results from the suggested method and those from the comparison method^23^ adopting the Student’s *t*-test and the variance ratio *F*-test^42^. The comparison method was an isocratic HPLC method using C18 column with mobile phase consisting of methanol: sodium phosphate dibasic anhydrous buffer (65:35 v/v) at pH of buffer 7.0 ± 0.02. It was found that the calculated values for Student’s *t*-test and the variance ratio *F*-test were less than the tabulated values. The obtained results were summarized in Table 2. The results proved that there was no significant difference between the performance of both methods in terms of accuracy and precision, respectively. The proposed liquid chromatographic method is characterized over the comparison reference method by its shorter time of elution. Moreover, using lower percentage of green organic solvent in the mobile phase is advantageous over classical mobile phases, as it has lower toxicity. Also, application of FFD abridged the traditional steps which consequently led to a reduction in consumption of time and chemicals.

### Precision

Two-levels precision was performed on each drug to evaluate the suggested technique precision. The repeatability of the proposed method was achieved by the determination of three different concentrations of studied drugs in raw materials on three successive time intervals. The inter-day precision was assessed through determination of the same concentrations in three successive days. The precision of the proposed method was confirmed by low values of % RSD and % Error, as shown in Table 3.

**Table 3:** Evaluation of intra- and inter-day precision of the analytical procedure of the studied drugs (50, 5.0, 5.0 and 10 μg/mL of PAR, LVC, PHN and AMB, respectively).

Drug	% Recovery* ± RSD	
	Intra-day precision	Inter-day precision
PAR	100.87 ± 0.61	100.05 ± 1.07
LVC	101.13 ± 0.78	100.30 ± 1.64
PHN	99.14 ± 1.03	99.44 ± 1.32
AMB	100.57 ± 1.20	100.95 ± 0.88

### Robustness

The robustness of the suggested method was confirmed by the steadiness of the peak areas upon minor alteration of different experimental parameters. Those alternations were carried out separately. The investigated variables were pH of the mobile phase (6.5 ± 0.1), ethanol percentage (20 ± 1%) and acetonitrile concentration (5 ± 0.5%). As shown in Table 4, no significant effect on the peak areas of the studied drugs was observed upon making these changes. Very minor or almost no change in the retention time was observed upon these minor changes in the optimum conditions. Based on that, the proposed method was proven to be robust.

**Table 4:** Robustness of the suggested technique using (50, 5.0, 5.0 and 10 μg/mL of PAR, LVC, PHN and AMB, respectively).

Parameter	Concentration found (µg/mL)				% Found			
	PAR	LVC	PHN	AMB	PAR	LVC	PHN	AMB
Ethanol ratio, %								
19	49.571	4.963	4.998	10.249	99.14	99.26	99.96	102.49
20	49.973	5.017	5.010	10.018	99.95	100.35	100.20	100.18
21	50.561	4.900	5.055	10.220	101.12	97.99	101.10	102.20
Mean					100.07	99.20	100.42	101.63
± S.D					1.00	1.18	0.60	1.26
%RSD					1.00	1.19	0.60	1.24
%Error					0.57	0.69	0.34	0.71
Acetonitrile ratio, %								
4.5	48.953	5.081	4.926	9.903	97.91	101.61	98.52	99.03
5.0	49.973	5.017	5.010	10.018	99.95	100.35	100.20	100.18
5.5	50.190	5.139	5.080	10.128	100.38	102.77	101.60	101.28
Mean					99.41	101.58	100.10	100.17
± S.D					1.32	1.21	1.54	1.13
%RSD					1.33	1.19	1.54	1.12
%Error					0.77	0.69	0.89	0.65
pH								
6.4	49.015	5.041	4.962	10.128	98.03	100.83	99.25	101.28
6.5	49.973	5.017	5.010	10.018	99.95	100.35	100.20	100.18
6.6	49.200	4.997	5.042	9.961	98.40	99.95	100.83	99.61
Mean					98.79	100.38	100.09	100.36
± S.D					1.02	0.44	0.80	0.85
%RSD					1.03	0.44	0.80	0.85
%Error					0.59	0.25	0.46	0.49

### System suitability

System suitability parameters were calculated referring to the USP^5^ and ICH Guidelines^31^ on a mixture of the four studied drugs. The obtained parameters were presented in Table 5.

**Table 5:** Parameters of system suitability of the proposed HPLC method for the determination of the studied drugs using 50 µg/mL synthetic mixture chromatogram.

Parameter	PAR	LVC		PHN		AMB
No of theoretical plates, N	1971	2567		6615		7744
Resolution, R_s_	3.87		7.7		5.1	
Retention time (t_R_), min	2.2	3.8		6.6		8.8
Tailing factor (T_f_)	1.13	1.01		1.12		1.17

### Selectivity and Specificity

The proposed method could determine the four studied drugs in presence of each other. Moreover, the specificity of the proposed method was assessed by observing any interference resulted from the used common excipients such as talc powder, maize starch, lactose, glucose and fructose (Table 6). The additives present in Panadol advance, Levcet and Ambroxol tablets also did not show any interference with the results (Table 7).

**Table 6:** Assay results for the determination of four studied drugs in their co-formulated prepared tablet by the suggested HPLC technique.

Ratio	Suggested technique												Comparison method^23^			
	Concentration taken (µg/mL)				Concentration found (µg/mL)				% Found				% Found			
	PAR	LVC	PHN	AMB	PAR	LVC	PHN	AMB	PAR	LVC	PHN	AMB	PAR	LVC	PHN	AMB
65:1:1:6	325.0	5.0	5.0	30.0	320.886	5.064	4.953	30.457	98.73	101.28	99.06	101.52	101.93	98.90	101.92	98.58
	390.0	6.0	6.0	36.0	397.759	5.901	6.051	35.208	101.99	98.35	100.85	97.8	99.54	101.21	98.43	101.50
	455.0	7.0	7.0	42.0	451.271	7.043	6.981	42.361	99.18	100.61	99.73	100.86	100.12	99.58	100.49	99.49
Mean									99.97	100.08	99.88	100.06	100.53	99.19	100.28	99.85
± S.D									1.77	1.54	0.91	1.99	1.25	1.19	1.754	1.49
*t-*test									0.45	0.16	0.35	0.075	The tabulated *t* and* F* values are 2.78 and 19.00, respectively at P = 0.05^42^			
*F*-test									2.01	1.68	3.76	1.76

**Table 7:** Assay results for the determination of some of the studied drugs in their single dosage form by the suggested HPLC technique.

Drug	Concentration taken (µg/mL)			Concentration found (µg/mL)			% Found		
	PAR in Panadol advance (0.5gm/tablet)	LVC in Levcet (5 mg/tablet)	AMB in Ambroxol (30 mg/tablet)	PAR	LVC	AMB	PAR	LVC	AMB
	175.0	2.5	30.0	177.319	2.445	29.934	101.33	97.8	99.78
	260.0	5.0	45.0	255.921	5.08	45.156	98.43	101.6	100.35
	350.0	7.0	60.0	351.887	6.96	59.915	100.54	99.43	99.86
Mean							100.01	99.61	99.99
± S.D							1.499	1.906	0.309

## Applications

The proposed technique was employed effectively to analyze PAR, LVC, PHN and AMB simultaneously in their raw materials, synthetic mixtures, combined prepared tablet and pharmaceutical dosage forms.

### Analysis of the studied drugs in raw material and synthetic mixtures

Simultaneous analysis of PAR, LVC, PHN and AMB in pure form and synthetic mixtures was carried out using the proposed HPLC method. The results were found to be in good agreement with those given using the comparison HPLC method^23^. The results abridged in Tables 2 and 8 indicate good recoveries of the studied drugs. Figure 3 represents good, resolved peaks of the four drugs in their laboratory-prepared mixture. Figure S4 illustrated a typical chromatogram of the laboratory-prepared synthetic mixture with the ratio of the marketed tablet.

**Table 8:** Assay results for the determination of the studied drugs in synthetic mixtures of their pharmaceutical ratio (65:1:1:6) by the suggested HPLC technique*.*

Ratio	Suggested technique											
	Concentration taken (µg/mL)				Concentration found (µg/mL)				% Found			
	PAR	LVC	PHN	AMB	PAR	LVC	PHN	AMB	PAR	LVC	PHN	AMB
65:1:1:6	325.0	5.0	5.0	30.0	324.499	5.039	5.051	30.296	99.85	100.78	101.02	100.99
	390.0	6.0	6.0	36.0	390.996	5.944	5.9	35.683	100.26	99.07	98.33	99.12
	455.0	7.0	7.0	42.0	454.505	7.023	7.05	42.119	99.89	100.33	100.71	100.28
Mean									100.00	100.06	100.20	100.13
± S.D									0.226	0.886	1.47	0.944

### Analysis of the studied drugs in the prepared tablet and pharmaceutical preparations

Table 6 represents the analysis of the studied drugs in their prepared tablet using the proposed method. These results were matched with those obtained by the comparison method^23^. Figure S5 illustrates the chromatogram of the studied drugs in their prepared tablet without interference of any additives. Statistical analysis using Student's t-test and variance ratio F-test^42^ revealed that there is no significant difference regarding accuracy and precision, respectively.

Table 7 showed the results of analysis of PAR, LVC and AMB in their single pharmaceutical preparations. The mean of percentage found was close to the true value and the standard deviation was less than 2, indicating accuracy and precision^31^ of the proposed method in analysis of the studied drugs in their dosage forms.

### Greenness estimation

Although the studies focused on eliminating the waste and adopting ecofriendly and sustainable methods^43^ were started in 1995, they were not assessed by the analytical society. One of the priorities of green analysis is to reduce the use of harmful substances without affecting the efficiency of the chromatographic performance^44^. The usage of environmentally friendly solvents in the mobile phase is one of the most important ways to obtain greener analysis^45^. The goal of this work is to declare that traditional dangerous techniques can be replaced by ecofriendly ones, while maintaining the same analytical behavior.

Recently, green analysis as well as indexing the method greenness have become very important. Indexing the method greenness allows the possibility of ranking the methods according to their greenness^46^. Four assessing methods were employed to assess the greenness of the recommended technique and compare it with reported ones.

First, the National Environmental Methods Index (NEMI) was applied on the proposed and reported methods. NEMI is a tool that uses the greenness profile and is regarded as one of the first methods to be used by researchers^27^. Table 9 showed that the proposed method achieved the four criteria of the greenness profile and is greener than the reported HPLC methods, according to NEMI profile. Water and ethanol are neither classified as PBT nor hazardous by the EPA’s Toxic Release Inventory^43,47^. The pH (6.5) of the mobile phase is not corrosive and the waste is less than 50 g/run.

Second, GAPI (Green Analytical Procedure Index)^46^ was also applied on the proposed and reported methods. Complexed GAPI was presented by Justyna Płotka-Wasylka and Wojciech Wojnowski and they also provide a very helpful software to automatically generate the complexed GAPI figure very easily depending on the inputs of each method^48^. The green assessment using that software for the proposed and reported HPLC methods was presented in Table 9.

Additionally, analytical Eco-scale was utilized for evaluating the proposed and reported methods, as represented in Tables 9 and 10. The score of the proposed method was 89, which referred to an excellent green methodology (the closer the score to 100, the greener the method)^49^.

**Table 9:** Comparison of sensitivity and greenness report between the suggested and reported HPLC methods.

Method	Linearity range μg/mL	Studied drugs	Mobile phase	Run time (min.)	Flow rate (mL/min.)	Waste (g/run) Run time × flow rate^10^	NEMI	Eco-scale score	Complex GAPI	AGREE
Reported HPLC method ^16^	5 to 30 diethyl-carbamazine 0.1 to 1 LVC	diethylcarbamazine and LVC	potassium dihydrogen orthophosphate buffer (pH: 3.2): acetonitrile (50:50 v/v)	6	1	6		66		
Reported HPLC method ^17^	5–35 for all the drugs	Salbutamol sulfate, Guaifenesin, and AMB	potassium dihydrogen ortho-phosphate buffer and methanol (58:42 v/v), pH 4.5	7	1	7		70		
Reported HPLC method ^18^	2.5–10 PHN, 250–750 PAR, 15–45 caffeine and 1.25–3.54 LVC	PHN, PAR, LVC and caffeine	Three steps gradient elution, 10 mM phosphate buffer (pH 3.3) and methanol 2–80%	18	1	18		67		
Reported HPLC method ^19^	50–150% for all the drugs	Ascorbic acid, PHN, PAR and LVC	A gradient elution using phosphate buffer pH 4.0: Acetonitrile	23	1.5	34.5		64		
Reported HPLC method ^20^	Not available	chlorpheniramine maleate, PAR and PHN	acetonitrile: a buffer solution of 0.01 M dipotassium phosphate and monopotassium phosphate (50:50), pH 7.8	14	1	14		65		
Reported HPLC method^22^	250–750 PAR, 1.25–3.75 LVC, 2.5–7.5 PHN and 30–90 AMB	PAR, LVC, PHN and AMB	Acetonitrile and Phosphate buffer, pH 3.5, three steps gradient elution	17.9	1.0	17.9		76		
Reported HPLC method^23^	162.5–975 PAR, 2.5–15 LVC, PHN and 15–90 AMB	PAR, LVC, PHN and AMB	Methanol: Sodium Phosphate dibasic anhydrous Buffer (65:35 V/V), pH of buffer 7.0 ± 0.02	12.3	1.0	12.3		79		
Proposed HPLC method	50 -500 PAR 0.5–20 LVC, PHN and 0.5–100 AMB	PAR, LVC, PHN and AMB	Ethanol: acetonitrile: 2.5 mM HSA (20:5:75), pH 6.5	9	1.0	9		89

**Table 10:** Eco-scale penalty points for the reported and the proposed HPLC methods.

Reported HPLC method^22^		Reported HPLC method^23^		Suggested HPLC method	
Reagents	Penalty points	Reagents	Penalty points	Reagents	Penalty points
Acetonitrile, 50 mL	4*3 = 12	Methanol, 65 mL	6*2 = 12	Ethanol, 20 mL	0*2 = 0
Phosphate buffer	0	Sodium Phosphate dibasic anhydrous Buffer	0	Acetonitrile, 5 mL	4*1 = 4
				HSA	0
∑	12		12		4

Instruments	Penalty points	Instruments	Penalty points	Instruments	Penalty points
HPLC–DAD	1	HPLC–UV	1	HPLC–UV	1
Occupational hazard	3	Occupational hazard	0	Occupational hazard	0
Waste, 18 mL No treatment	5 3	Waste, 13 mL	5 3	Waste,9 mL	3 3
	∑ 12		∑ 9		∑ 7
Total penalty points Score	24 100–24 = 76	Total penalty points Score	21 100–21 = 79	Total penalty points Score	11 100–11 = 89

Finally, the greenness of the proposed method was investigated using AGREE-Analytical Greenness Metric Approach and software through evaluating 12 parameters of green analytical aspects. The assessment result is in the form of a circle divided into twelve parameters with different colors ranging from dark green to orange, based on information reported by Francisco Pena-Pereira et al.^50^. The score was found to be 0.87 indicating the greenness of the method (the closer the score to 1.0 the greener the method). The AGREE results for the proposed and reported HPLC methods were presented in Table 9.

Based on the above results by the four assessment tools, it was concluded that the suggested HPLC technique has an environmental advantage over the two reported methods, and thus it could be employed for the routine analysis of the cited drugs without affecting the environment.

### Comparison of developed and published methods

The proposed method was compared with the two reported HPLC methods that were used for the determination of the same quaternary mixture. It was found that the proposed method was more sensitive, simple, green and need shorter time than the two reported method, as illustrated in Table 9. Regarding sensitivity, from the illustrated values of the linearity range it was found that the suggested method was more sensitive than the two reported methods. In addition, they show reasonably wider linearity ranges compared to other methods. Regarding the simplicity in performing the chromatographic conditions, the developed method represents the simplest and most economic method, as it consumes the lowest volume of mobile phase and therefore generates the lowest volume of waste. The first published method^22^ was based on gradient elution and DAD- detector, which is not available at all labs. In addition, gradient elution consumes large amount of the used solvents (acetonitrile). The second published method^23^ was based on isocratic elution- UV detector with mobile phase consisting of large amount of methanol (65 mL). The four drugs needed 18 min. for separation by first method and 13 min. by the second one, while 9 min. by the proposed method.

## Conclusion

The current study presented a green, simple, rapid, direct, sensitive with wide range of linearity HPLC method for the assay of a drug combination consisting of PAR, LVC, PHN and AMB, typically indicated for relief of cold symptoms. All the four analytes were successfully resolved and quantified in a relatively short run time (within 9 min.) using the least possible amount of organic solvents (20 % ethanol, 5 % acetonitrile). The developed liquid chromatographic method has limit of detections (LOD) between 29 and 147 ng/mL and limit of quantification (LOQ) between 95 and 485 ng/mL. The proposed method was optimized and developed using a two-level FFD to predict the system suitability parameters. Employing FFD participated in decreasing the chemicals consumption, analysis steps and time. The recommended mobile phase was mainly selected for substituting unsafe solvents (such as large amounts of methanol and acetonitrile) without influencing the chromatographic performance in order to make it eligible for routine analysis. From an economical point of view, the method involved inexpensive analytical reagents which are available in any analytical laboratory. The recommended technique was found to have low environmental impact which was ensured by investigating the method’s greenness using four assessment tools. It was successfully applied for analysis of the four drugs either in synthetic mixture or combined prepared tablets. The reasonable mean recovery percentage of tablet forms (between 99.61 and 100.08 ± 0.31 to 3.76) proved that the excipients have no interference in the determination.

## Supplementary Information

Supplementary Figures.

## Data Availability

The data that support the findings of this study are available on request from the corresponding author [N. A.].

## Abbreviations

UV-HPLC: Ultra-violet High-performance liquid chromatography
PAR: Paracetamol
LVC: Levocetirizine dihydrochloride
PHN: Phenylephrine hydrochloride
AMB: Ambroxol hydrochloride
NEMI: National Environmental Methods Index
GAPI: Green Analytical Procedure Index
AGREE: Analytical greenness metric
NSAIDs: Non-steroidal anti-inflammatory drugs
USP: United States Pharmacopeia
BP: British Pharmacopeia
JP: Japanese pharmacopeia
CNS: Central nervous system
EP: European Pharmacopeia
DAD: Diode-Array Detection
HSA: Heptane-1-sulphonic acid sodium salt
DOE: Experimental design
FFD: Full factorial design
RP-HPLC: Reverse phase HPLC
t_R_: Retention time
pH: Potential of hydrogen
k: Number of independent factors
m: Level combinations
ACN: Acetonitrile
T_f_: Tailing Factor
Rs: Resolution
ICH: International Conference on Harmonization Guidelines
LOD: Limit of detection
LOQ: Limit of quantitation
S. D: Standard deviation
RSD: Relative standard deviation
