# Supplementary material for: A quality-by-design eco-friendly UV-HPLC method for the determination of four drugs used to treat symptoms of common cold and COVID-19
Source: Sci Rep. 2023 Jan 28;13:1616. doi: 10.1038/s41598-023-28737-3 (PMC9884070; doi:10.1038/s41598-023-28737-3)
Supplement: Supplementary file 1 — Supplementary Figures. [file 41598_2023_28737_MOESM1_ESM.docx]

**Supplementary Material**

**A Quality-by-Design Eco-friendly UV-HPLC Method for the Determination of Four Drugs Used to treat Symptoms of Common Cold and COVID-19**

Nora A. Abdallah^*^, Mona E. Fathy, Manar M. Tolba, Amina M. El-Brashy and Fawzia A. Ibrahim

Department of Pharmaceutical Analytical Chemistry, Faculty of Pharmacy,

Mansoura University, Mansoura 35516, Egypt.

*Corresponding author.

Tel.: +20502246253, Fax: +20502363641, E-mail: [noraabdallah91@mans.edu.eg](mailto:noraabdallah91@mans.edu.eg)

**Figure S1:** 2^3^ FFD pareto charts of the effects on the chromatographic responses at alpha = 0.05.

**Figure S2, 3:** 2^3^ FFD main effect & full interaction plots for chromatographic responses by data means type.

**Figure S4:** Typical chromatogram of PAR, LVC, PHN and AMB laboratory synthetic mixture (65:1:1:6) under the described chromatographic conditions

**Figure S5:** Typical chromatogram of PAR, LVC, PHN and AMB laboratory prepared tablet under the described chromatographic conditions


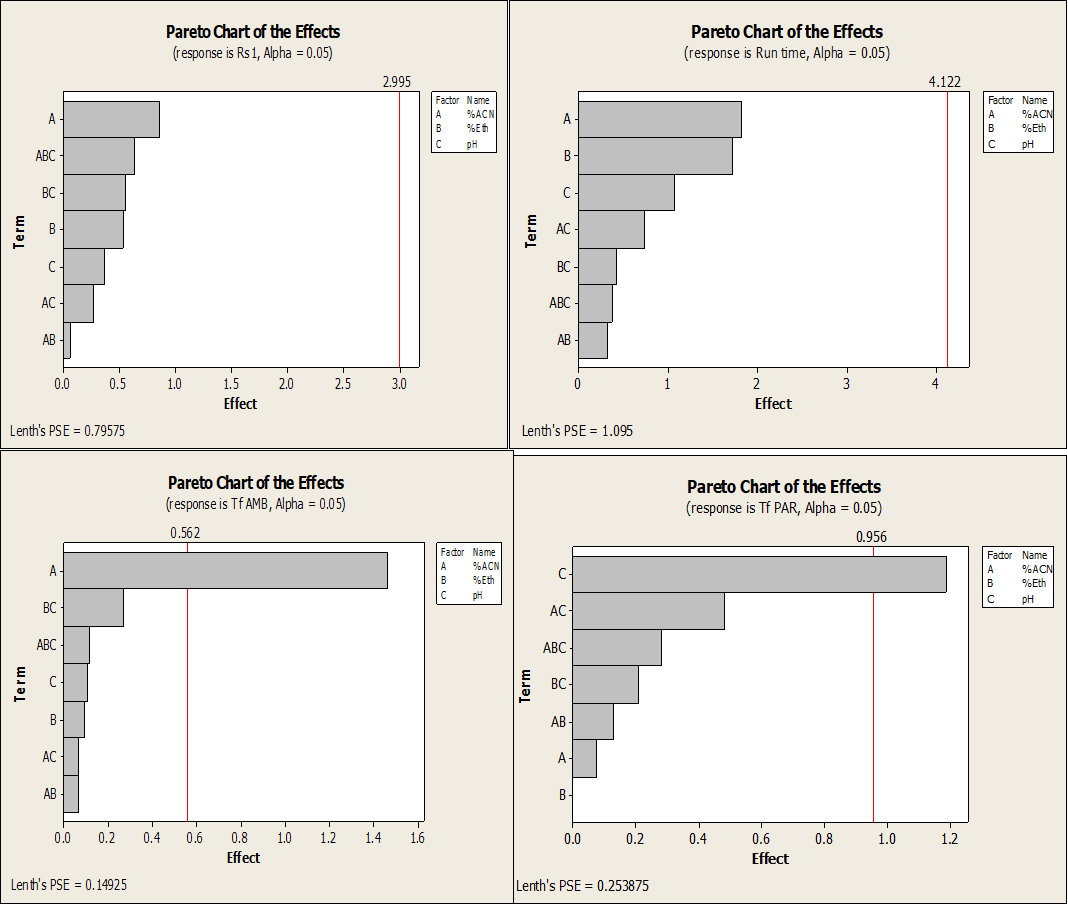


**Figure S1**

**Figure S2**

**Figure S2**

**Figure S3**


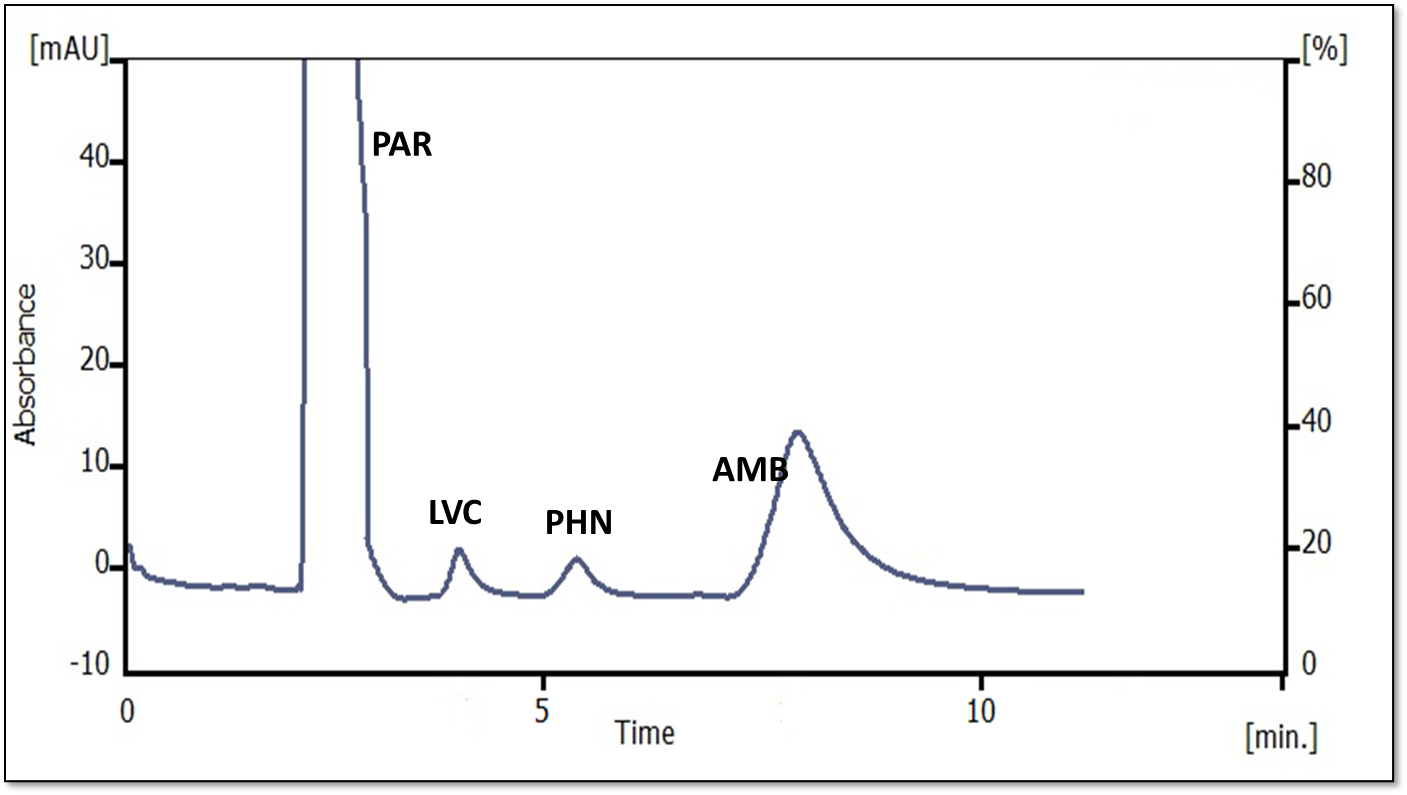


**Figure S4**


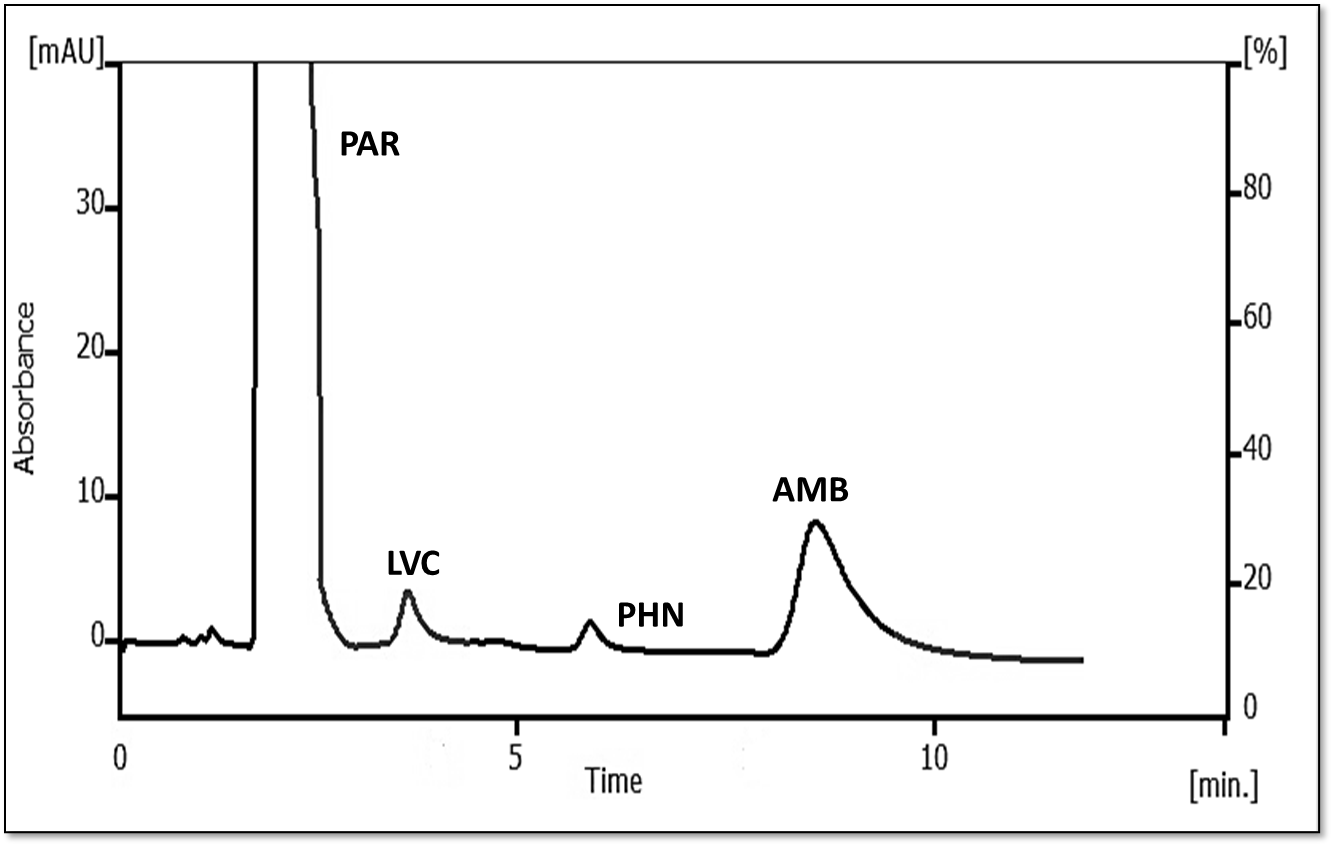


**Figure S5**
